# Combining Comprehensive Analysis of Off-Site Lambda Phage Integration with a CRISPR-Based Means of Characterizing Downstream Physiology

**DOI:** 10.1128/mBio.01038-17

**Published:** 2017-09-19

**Authors:** Yu Tanouchi, Markus W. Covert

**Affiliations:** Department of Bioengineering, Stanford University, Stanford, California, USA; Korea Advanced Institute of Science and Technology

**Keywords:** CRISPR/Cas 9, DNA sequencing, phage lambda

## Abstract

During its lysogenic life cycle, the phage genome is integrated into the host chromosome by site-specific recombination. In this report, we analyze lambda phage integration into noncanonical sites using next-generation sequencing and show that it generates significant genetic diversity by targeting over 300 unique sites in the host *Escherichia coli* genome. Moreover, these integration events can have important phenotypic consequences for the host, including changes in cell motility and increased antibiotic resistance. Importantly, the new technologies that we developed to enable this study—sequencing secondary sites using next-generation sequencing and then selecting relevant lysogens using clustered regularly interspaced short palindromic repeat (CRISPR)/Cas9-based selection—are broadly applicable to other phage-bacterium systems.

## IMPORTANCE

Bacteriophages play an important role in bacterial evolution through lysogeny, where the phage genome is integrated into the host chromosome. While phage integration generally occurs at a specific site in the host chromosome, it is also known to occur at other, so-called secondary sites. In this study, we developed a new experimental technology to comprehensively study secondary integration sites and discovered that phage can integrate into over 300 unique sites in the host genome, resulting in significant genetic diversity in bacteria. We further developed an assay to examine the phenotypic consequence of such diverse integration events and found that phage integration can cause changes in evolutionarily relevant traits such as bacterial motility and increases in antibiotic resistance. Importantly, our method is readily applicable to other phage-bacterium systems.

## OBSERVATION

Lysogeny—a natural part of the bacteriophage life cycle during which the phage chromosome is integrated into the host chromosome—plays an important role in the diversification of bacterial genomes. Bacterial genomes typically contain a variety of prophage elements, and ecologically important traits are carried on prophages in many bacterial species (1, 2). Notably, phage-derived virulence genes are often observed in the genome of bacterial pathogens, and the acquisition of such genes through lysogeny can transform nonpathogenic strains of bacteria into pathogens (1, 3). While lysogenic conversion, a process in which phages act as a vehicle for horizontal transfer, is widely recognized and has been extensively studied, the direct consequence of the phage integration that changes the host genetic structure is understudied and has begun to be appreciated only recently (1, 4). This is mainly due to the fact that, except for some transposable classes of phage (4), phage integration during lysogeny is generally believed to preserve (or compensate for the change in) the host genetic structure (5).

In the canonical model of lysogeny, phage integration is mediated by phage-encoded DNA recombinases and takes place at a specific attachment site in the host genome (*attB*) which shares high homology with its counterpart in the phage genome (*attP*) (6). While the recombination between these two sites is highly sequence specific, phage integration into secondary (i.e., non-*attB*) sites has been observed—albeit at a low frequency—in host bacteria that lack *attB* (7–9). Furthermore, phage integration into secondary sites has been shown to disrupt surrounding host genes and cause phenotypic changes such as auxotrophy (8, 9). Despite the obvious evolutionary implications of such events, little is known about the diversity of secondary sites and the physiological effects such integrations may impose on the host.

**FIG 1:**
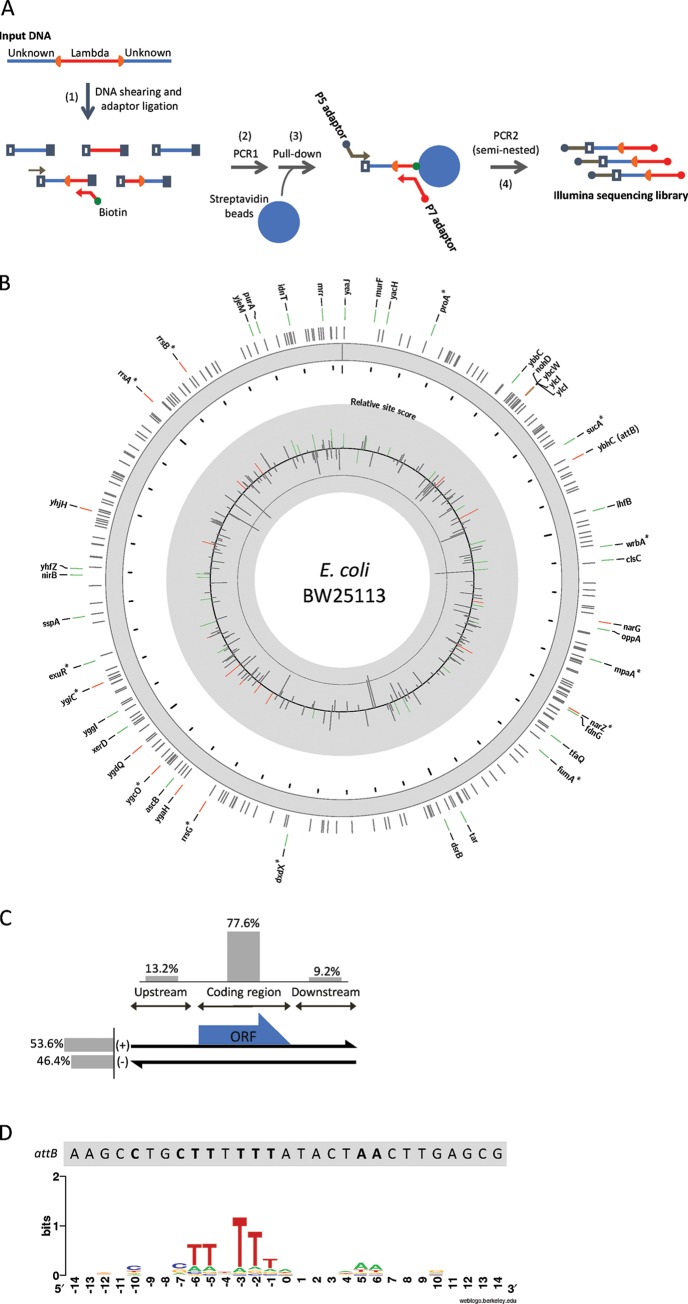
NGS analysis of phage integration sites. (A) A schematic diagram of NGS library preparation. Genomic DNA extracted from phage-infected *E. coli* (input DNA) is first enzymatically fragmented (step 1) and then ligated with adaptors (step 2). The fragments are PCR amplified with an adaptor-specific primer (gray) and a biotinylated primer specific to phage sequence (red) (PCR1) (step 3). The biotinylated PCR fragments are pulled down by magnetic streptavidin beads (step 4). Another round of PCR is performed using an adaptor-specific primer (gray) and phage-specific primer (red) containing the sequences necessary for Illumina sequencing. The phage-specific primer is internal to the primer used in PCR1, making this PCR seminested for increased specificity. (B) A genome-wide landscape of detected integration sites. On the outer ring, the positions of integration sites are shown. The color code indicates that the site was found in 1 (black), 2 to 4 (green), or >4 (red) replicate samples. For those that were found in multiple (i.e., green and red) replicate samples, the names of the genes closest to each integration site are shown. *, sites that were also found in the work described in references 7 and 9. The inner ring shows the motif scores of each integration site relative to the average score for all integration sites found. The dashed line indicates the average score of all possible 29-base genomic sequences. (C) A summary of the locations of the detected secondary integration sites. The secondary integration sites were categorized by strandness (left) or by their position with respect to host genes (top). The upstream and downstream positions are defined with respect to the gene closest to the integration site. (D) The sequence motif of integration sites. A total of 29 bases around each of the integration sites were inputted into the WebLogo program (http://weblogo.berkeley.edu/) to generate the motif. For comparison, the corresponding *attB* sequence is also shown (top); bold letters indicate bases that match the motif.

Using phage lambda and its host *Escherichia coli*, arguably the best-studied phage-bacterium infection model, we sought to characterize the genomic landscape of lambda secondary integration sites. To this end, we developed a next-generation sequencing (NGS) method to specifically sequence lambda-*E. coli* DNA junctions in a high-throughput manner (Fig. 1A). Our analysis of 8 replicate sequencing reactions (four technical replicates and two biological replicates) identified 304 unique secondary sites (Fig. 1B; see also Table S1 in the supplemental material), 290 of which were novel (in comparison to the results presented in references 7 and 9. A total of 77.6% of these sites fall within bacterial coding regions and thus likely disrupt gene expression (Fig. 1C). Also, the secondary sites are equally represented on the positive and negative strands of the genome, indicating that there is no strand bias for phage integration (Fig. 1C). Additionally, a recent study found that the frequency of lambda integration into *attB* is dependent on its location in the genome relative to the origin of replication (24). We did not observe a pattern of secondary sites that is consistent with this. Overall, the number of reads that were mapped to secondary sites was on average 0.51% of those that were mapped to *attB*. This is consistent with a previous finding that the frequency of phage integration in an *E. coli* strain deleted for *attB* is reduced about 100-fold to 1,000-fold relative to integration into wild-type *E. coli* (6). However, unlike previous studies, our method allowed detection of secondary sites without the need to delete *attB* from the host genome. Therefore, our result not only uncovered the large diversity of secondary sites but also suggests that phage integration into secondary sites is a more widespread and common phenomenon than previously believed.

10.1128/mBio.01038-17.3 TABLE S1  A complied list of phage integration sites identified by NGS. Download TABLE S1, XLSX file, 0.1 MB. Copyright © 2017 Tanouchi and Covert. 2017 Tanouchi and Covert This content is distributed under the terms of the Creative Commons Attribution 4.0 International license.

Phage integration is generally mediated by site-specific recombination (6). The sequence comparison of secondary sites revealed highly conserved thymine residues at the −1, −2, −3, −5, and −6 positions (Fig. 1D). Both the cytosine residues at − 7 and − 10 and the adenine residues at +5 and +6 are also well conserved. These sequence features are also present in the *attB* site and are consistent with the results of a previous study that examined a smaller set of secondary sites in phage lambda (9) under similar conditions. The existence of conserved sequence features suggests that phage integration into secondary sites is mediated by the same mechanism as integration into *attB*. Using this sequence motif, we computed a position-specific scoring matrix and scored each secondary site according to its similarity to the motif. We found that the integration sites that were found across multiple replicate samples scored more highly on average than those found in a single sample (see Fig. S1A in the supplemental material), providing further support for the idea that sequence elements represent the major determinants of phage integration. Interestingly, when we computationally surveyed the host genome, we observed a significant number of sites that have a high score but that were not detected by our NGS assay (Fig. S1B). While our list of integration sites is likely incomplete, this observation may also indicate that unknown host, phage, or environment factors play a role in determining the spectrum of phage integration sites.

10.1128/mBio.01038-17.1 FIG S1  Analysis of sequence score. (A) The integration sites were grouped by detection frequency (the number of replicate samples that detected the site of interest), and their average scores (calculated based on the motif in Fig. 1D) are shown (red line). The number of integration sites that fall into each group is also shown (gray bar). (B) The distributions of sequence scores for the detected integration sites (red) and all 29-base sequences on the host genome (gray) are shown. Download FIG S1, EPS file, 0.2 MB. Copyright © 2017 Tanouchi and Covert. 2017 Tanouchi and Covert This content is distributed under the terms of the Creative Commons Attribution 4.0 International license.

**FIG 2:**
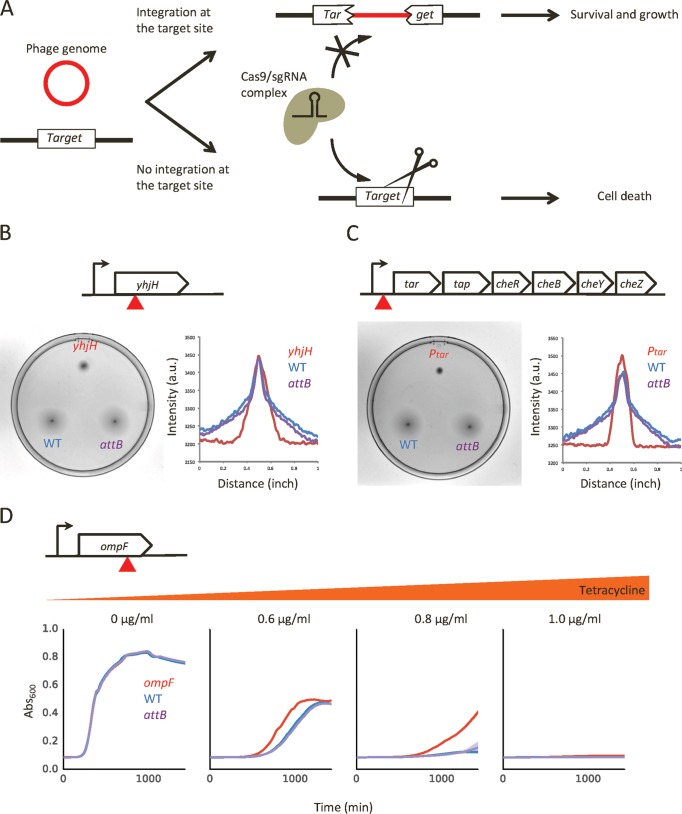
Analysis of lysogens with integration into secondary sites. (A) A schematic diagram illustrating the CRISPR/Cas9-based strategy for selection of lysogens of interest. The use of a Cas9/sgRNA complex targeting a specific target site allows cell survival and growth only when the target site is disrupted by phage integration. (B) Swimming assay for the *yhjH* lysogen. The top diagram shows the location of the phage integration site (red triangle). As controls, the wild-type (WT) strain (BW25113; blue) and *attB* lysogen (lysogen that has phage integration into *attB* but not into *yhjH*, *P*_*tar*_, or *ompF* [Fig. S2A]; purple) were used. The swimming assay plate was imaged (left), and the cross section of growth area was analyzed (right). a.u., absorbance units. (C) Data were determined as described for panel B but represent the *P*_*tar*_ lysogen. (D) Growth assay of the *ompF* lysogen with increasing concentrations of tetracycline. The top diagram shows the location of phage integration (red triangle). Each growth curve represents a value averaged from results from four technical replicates, and the shaded region represents the standard deviation.

The finding that nearly 80% of secondary sites occur within an open reading frame (ORF) prompted us to examine the effects of secondary integration on host physiology. Such an investigation would typically require screening a large number of colonies to isolate lysogens that have phage integrated at a site of interest (9). This would be particularly challenging and prohibitively labor intensive in using *E. coli* strains with an intact *attB* site (e.g., wild-type strains) where the frequency of phage integration into *attB* far exceeds the frequency of phage integration into secondary sites. To overcome this challenge, we devised a novel clustered regularly interspaced short palindromic repeat (CRISPR)/Cas9-based selection method (10, 11) to isolate lysogens that have lambda integration at a specific site (Fig. 2A). Briefly, we designed a single guide RNA (sgRNA) that targets the integration site of interest in the host genome. In lysogens, expression of sgRNA and Cas9 leads to cleavage of the host genome and cell death—unless the target site has been disrupted by phage integration. Thus, our method enables rapid selection for cells that have phage integrated at a specific target site. The isolated lysogens can then be used for phenotypic assays. We applied this method to examine the effect of phage integration into three of our newly discovered sites: *yhjH*, the promoter region of *tar* (*P*_*tar*_), and *ompF*. Both *yhjH*, encoding a c-di-GMP phosphodiesterase (12, 13), and *P*_*tar*_ a promoter driving the core components of the chemotaxis signaling pathway (14, 15), are directly involved in the regulation of cell motility. *ompF* codes for an outer membrane porin that mediates transport of various solutes. We note that these three examples represent sites that were detected at different frequencies in the NGS experiment; *yhjH*, *P*_*tar*_, and *ompF* were found in 5, 2, and 1 replicate samples, respectively (red, green, and black bars, respectively, in Fig. 1B). Regardless of the detection frequency, we were able to successfully isolate the lysogens, and we confirmed the expected phage integration (i.e., the presence of a lambda-*E. coli* DNA junction that is consistent with the NGS result) into these genomic locations by PCR and Sanger sequencing (Fig. S2). Moreover, we found that these lysogens also had phage integrated at *attB* (Fig. S2A). In our experiment, most host cells were infected with multiple phages (i.e., high average phage input [API]; see Materials and Methods). As recent studies showed that coinfecting phages execute lysogeny in a cooperative manner (25, 26), it would be interesting to see how the locations of integration sites are determined among coinfecting phages.

10.1128/mBio.01038-17.2 FIG S2  Validation of phage integration into *ompF*, *P*_*tar*_, and *yhjH* sites. (A) PCRs were performed with primers designed to specifically detect an *E. coli*-lambda junction at the *ompF*, *P*_*tar*_, *yhjH*, or *attB* site. We confirmed that (i) the *attB* lysogen does not carry phage integration into the *ompF*, *P*_*tar*_, or *yhjH* sites and (ii) the *ompF*, *P*_*tar*_, and *yhjH* lysogens all contain phage integration into their respective sites. We also observed that these lysogens also carry phage integration into *attB*. (B) The *E. coli*-lambda junction was PCR amplified and sequenced by Sanger sequencing to confirm proper integration in *ompF*, *P*_*tar*_, and *yhjH* lysogens. Download FIG S2, EPS file, 1.8 MB. Copyright © 2017 Tanouchi and Covert. 2017 Tanouchi and Covert This content is distributed under the terms of the Creative Commons Attribution 4.0 International license.

The deletion of *yhjH* and the chemotaxis operon have been shown to reduce swimming ability (14, 16), and we hypothesized that phage integration into *yhjH* and *P*_*tar*_ sites would exhibit a similar effect. Indeed, when *yhjH* and *P*_*tar*_ lysogens were spotted on soft agar, they showed impaired swimming motility compared to control strains (Fig. 2B and C). This change in cell motility may have important evolutionary implications; as an example, the loss of motility has been shown to be a characteristic adaptive trait in *Pseudomonas aeruginosa* during chronic respiratory infection and biofilm growth (4, 17, 18). The deletion of *ompF* has been shown to increase tetracycline resistance by slowing down the influx of tetracycline molecules into the cell (19, 20). Consistent with this function, when the *ompF* lysogen was grown using various tetracycline concentrations, we observed elevated drug resistance (Fig. 2D). This result illustrates a phage-based mechanism of antibiotic resistance that emerges without involving the transfer of antibiotic resistance genes.

The three cases that we examined thus represent clear examples of how phage integration into secondary sites can cause evolutionarily relevant changes in bacterial physiology. However, the complete list of our newly found integration sites includes many other sites that might affect various cellular processes such as metabolism, respiration, and transport (Table S1). For example, there are 9 genes involved in nitrate respiration that are disrupted by phage integration. Our CRSIPR/Cas9-based selection approach can be easily extended to study these sites provided that an appropriate protospacer adjacent motif (PAM) sequence exists near the site of interest. Another interesting set of sites was found just upstream of certain rRNA operons (*rrsA*, *rrsB*, *rrsC*, and *rrsG*). rRNA operons usually contain tRNA genes, and many temperate phages are known to contain various tRNA genes (21); therefore, while the evolutionary significance of having tRNA genes on phage genome is still actively debated, phage integration into secondary sites and subsequent prophage induction might represent potential mechanisms by which phages obtain tRNA genes. Unfortunately, isolating these lysogens using our CRISPR/Cas9-based selection method is complicated by the existence of high sequence homology among rRNA operons and the associated difficulty in designing an sgRNA unique to a particular rRNA operon. Finally, it is interesting that recent studies suggested that some prophages found within a host gene can act as an active “switch” to control host physiology (22, 23); in the presence of appropriate environmental stimuli, the phage excises its genome and consequently restores the host gene to allow its expression. It is tempting to speculate on a potential connection between secondary integration sites and the evolution of such prophage-based switches.

The generation of genetic diversity is a key ingredient of evolution. In this context, bacteriophages are conventionally seen as a vehicle for horizontal gene transfer or as a “genetic reservoir” that is accessible to bacterial populations. In this study, we revisited the classical model of lysogeny in the lambda-*E. coli* system to take a closer look at phage integration into secondary sites. Our NGS strategy allowed detection of phage integration in the presence of the *attB* site and revealed that lambda targets over 300 unique sites in the host genome for integration. This result suggests that phage integration may be a general mechanism for the genomic diversification that drives bacterial evolution. Indeed, the characterization of lysogens that have phage integrated at three distinct sites illustrated that phage integration into secondary sites led to changes in cell motility and antibiotic resistance of the host, both of which are evolutionarily relevant. Importantly, the new framework that we developed to enable this study—sequencing secondary sites using NGS and then selecting relevant lysogens using CRISPR/Cas9-based selection—is broadly applicable to other phage-bacterium systems. Looking forward, most bacteria live in a complex environment that dynamically changes over time and are in constant contact with phages. More work is needed to understand the full scope of the effects that phage integration can exert on the host and how it shapes bacterial evolution.

### Strains, plasmids, and primers.

Wild-type lambda (ATCC 23724-B2) was used throughout the study, and *E. coli* BW25113 was used as a host strain. Phage particles were purified by ultracentrifugation with a CsCl step gradient and then with an equilibrium gradient per the standard protocol. All plasmids (Table S2) were constructed with standard molecular biology techniques. In Fig. 2B to D, the “*attB*” strain data represent a lysogen with confirmed phage integration into *attB* but not into *yhjH*, *P*_*tar*_, or *ompF* (Fig. S2A). The primers used for NGS library preparation are listed in Table S3.

10.1128/mBio.01038-17.4 TABLE S2  Plasmid list. Download TABLE S2, XLSX file, 0.03 MB. Copyright © 2017 Tanouchi and Covert. 2017 Tanouchi and Covert This content is distributed under the terms of the Creative Commons Attribution 4.0 International license.

10.1128/mBio.01038-17.5 TABLE S3  Primer list. Download TABLE S3, XLSX file, 0.02 MB. Copyright © 2017 Tanouchi and Covert. 2017 Tanouchi and Covert This content is distributed under the terms of the Creative Commons Attribution 4.0 International license.

### Phage infection and DNA extraction.

An overnight culture of strain BW25113 grown in LB at 37°C was diluted 100-fold into fresh LBMM (LB medium supplemented with 10 mM MgSO_4_ and 0.2% maltose) and cultured at 37°C for 3 h. The cells were spun down and resuspended in 10 mM MgSO_4_ to an optical density at 600 nm (OD_600_) of 2.0 (∼10^9^ cells/ml). After incubation at 37°C for 1 h, the cells were put on ice for 10 min, 1 ml of cells and 1 ml of phage suspension were mixed to reach an average phage input (API) value of 10, and the reaction mixture was further incubated at 4°C for 30 min for adsorption. The mixture was then diluted 10-fold into warm LBMM and was cultured at 37°C. After 1 h, the culture was put on ice for 15 min and the genomic DNA was extracted using a DNeasy Blood and Tissue kit (Qiagen).

### NGS library preparation.

For each sequencing run, four technical replicates of DNA library were prepared. The overall workflow is shown in Fig. 1A. All PCRs were performed using Phusion polymerase (NEB). For each library, 50 ng of DNA extracted from phage-infected *E. coli* cells was tagmented using a Nextera DNA sample preparation kit (Illumina). The DNA was purified by the use of a DNA Clean and Concentrator-5 kit (Zymo) and subjected to a single-primer PCR with primer bio-attL (1 cycle at 98°C for 30 s; 12 cycles at 98°C for 10 s, 62 C for 30 s, and 72°C for 30 s; 1 cycle at 72°C for 5 min). The reaction mixture was spiked with Nextera P7-3′ primer and subjected to an additional 25 cycles of PCR (1 cycle at 98°C for 30 s; 25 cycles at 98°C for 10 s, 62°C for 30 s, and 72°C for 30 s; 1 cycle at 72°C for 5 min). The PCR product was purified by the use of a DNA Clean and Concentrator-5 kit (Zymo) and then pulled down using Dynabeads MyOne streptavidin C1 magnetic beads (Thermo Fisher Scientific) per the manufacturer’s protocol. Seminested PCR was performed on the magnetic beads with P7-N70X (× = 1 to 4) and P5-N501-N4-attL primers (1 cycle at 98°C for 30 s; 20 cycles at 98°C for 10 s, 62°C for 30 s, and 72°C for 30 s; 1 cycle at 72°C for 5 min). The PCR product was purified using a 0.6:1 ratio of Agencourt AMPure XP beads (Beckman Coulter, Inc.) to the sample. The resulting libraries were pooled at equimolar ratios and sequenced using 2 × 75-bp paired-end sequencing in MiSeq (Illumina).

### Sequencing analysis.

The NGS results were analyzed as follows. First, to ensure the analysis of actual phage integration events, the paired-end reads were filtered and only those that contained the expected phage sequence (5′-GTTGCAACAAATTGATAAGCAATGCTTTTTTATAATGCCAACTTA-3′) in read 1 without any mutation were selected. The phage sequence was subsequently removed, and the 3′ ends of the remaining reads were trimmed based on the quality score (minimum Phred score of 10 and average score of 15). Next, 7 bases of the 5′ end in read 1, which correspond to the region of strand exchange during phage integration, were trimmed. Read 1 reads whose lengths consisted of at least 16 bases and 20 bases at the 5′ end of read 2 were used to map onto the genome sequence of strain BW25113 (assembly, GCA_000750555.1) with a 95% identity cutoff value. The resulting sam file was analyzed using a custom python script to identify integration sites and their locations on the *E. coli* genome. The motif analysis was performed using the *motif* package in Biopython. Processing of sequencing files and mapping were done using the BBMap package (version 34.33). Raw sequence data are available from Sequence Read Archive (see below).

### CRISPR/Cas9-based selection of lysogens.

An overnight culture of strain BW25113 carrying pCas9 was diluted 100-fold in LBMM and grown at 30°C for 3 h. The cells were spun down and resuspended in 10 mM MgSO_4_ to an OD_600_ of 2.0 (∼10^9^ cells/ml). After incubation at 30°C for 1 h, the cells were put on ice for 10 min, 1 ml of cells and 1 ml of the phage suspension were mixed to reach an API of 10, and the reaction mixture was further incubated at 4°C for 30 min for adsorption. The mixture was then diluted 10-fold into warm super optimal broth (SOB; 2% tryptone, 0.5% yeast extract, 0.05% NaCl) containing 100 ng/ml anhydrotetracycline and was cultured at 30°C. After 2 h, the cells were put on ice for 10 min and made competent by 5 washes with ice-cold water. The cells were transformed with a guide RNA plasmid (pgRNA) that contains an sgRNA of interest (e.g., pgRNA-yhjH) by electroporation. The transformants were screened for proper phage integration by colony PCR. Finally, pCas9 and pgRNA plasmids were cured by culturing at 37°C.

### Swimming motility assay.

BW25113 cells grown overnight in TB (1% tryptone, 0.5% NaCl) at 30°C were diluted 100-fold into fresh TB and cultured for 4 h. A 1-µl volume of cells was inoculated into a 0.25% TB agar plate by stabbing. The plate was sealed with Parafilm and incubated at 30°C in an inverted position for 16 h. The plate was imaged using a Gel Doc EZ Imager (Bio-Rad) with 0.1 s of exposure.

### Tetracycline sensitivity assay.

BW25113 cells grown overnight in LB at 37°C were diluted 2 × 10^4^-fold into fresh LB containing various concentrations of tetracycline and transferred to a 96-well plate. Cells were then grown using an Infinite 200 Pro microplate reader (Tecan) with continuous shaking. The temperature was kept at 37°C, and cell growth was monitored by measuring absorbance at 600 nm approximately every 9 min. Each condition was represented by four technical replicates.

### Accession number(s).

Raw sequence data are available from the Sequence Read Archive under accession number SRP107822.
